# A Cross-Sectional Epizootiological Study and Risk Assessment of Foot-Related Lesions and Lameness in Intensive Dairy Sheep Farms

**DOI:** 10.3390/ani11061614

**Published:** 2021-05-29

**Authors:** Marios Moschovas, Aphrodite I. Kalogianni, Panagiotis Simitzis, Georgios Pavlatos, Stavros Petrouleas, Ioannis Bossis, Athanasios I. Gelasakis

**Affiliations:** 1 Laboratory of Anatomy and Physiology of Farm Animals, Department of Animal Science, School of Animal Biosciences, Agricultural University of Athens (AUA), Iera Odos 75 str., 11855 Athens, Greece; moschovas@aua.gr (M.M.); afrokalo@aua.gr (A.I.K.); pavlatos1996@gmail.com (G.P.); stud214096@aua.gr (S.P.); 2 Laboratory of Animal Breeding and Husbandry, Department of Animal Science, School of Animal Biosciences, Agricultural University of Athens (AUA), Iera Odos 75 str., 11855 Athens, Greece; pansimitzis@aua.gr; 3 Laboratory of Animal Husbandry, Department of Agricultural Sciences, School of Agriculture, Forestry and Natural Resources, Aristotle University of Thessaloniki (AUTH), 54124 Thessaloniki, Greece; bossisi@agro.auth.gr

**Keywords:** lameness, dairy sheep, intensive system, infectious footrot, white line disease, ovine interdigital dermatitis, hoof overgrowth, risk factors, sheep welfare

## Abstract

**Simple Summary:**

Foot-related lameness is one of the most significant welfare issues in farm animals. Contrary to dairy cows and meat sheep breeds, epizootiological data on foot-lesions and associated lameness in dairy sheep are scarce. In this study, data were collected from 30 representative intensive dairy sheep farms. Multivariate statistical analysis was used to produce a typology of intensive farming systems which resulted in the assignment of farms in two distinct clusters. Six hundred adult ewes were randomly selected from six flocks (three flocks per cluster) and a cross-sectional study was implemented to investigate the epizootiology and potential risk factors of foot-related lameness, foot-lesions and diseases. Ovine interdigital dermatitis and infectious footrot were the most common infectious foot diseases, while white line disease and hoof wall cracks were the most prevalent non-infectious lesions. Infectious footrot was the main cause of lameness and increased with age, whereas body condition score was associated with increased prevalence of ovine interdigital dermatitis. Comparisons between the clusters regarding foot-related lameness, foot-diseases and lesions at the animal, the limb, and the hoof level are presented, and relevant literature, mechanisms, hypotheses, and challenges of the field are discussed.

**Abstract:**

Foot-related lameness, foot-diseases and lesions are emerging issues in dairy sheep; however, relevant epizootiological studies are scarce, and risk factors have not been elucidated. The objectives of this cross-sectional study were (i) to address this dearth of knowledge by investigating the epizootiology of lameness-related foot-lesions and diseases, and (ii) to assess the impact of potential risk factors on foot health, in intensive dairy sheep farms. Thirty farms were assigned in two representative clusters using a multivariate statistical analysis. Three farms per cluster and 100 multiparous milking ewes per farm (total n = 600) were selected and enrolled in the study. Foot-related lameness, ovine interdigital dermatitis (OID), infectious footrot (IFR), white line disease, hoof wall cracks, as well as health and welfare traits were recorded. Overall prevalence of foot-related lameness was 9.0% and was primarily associated with IFR; however, additional infectious and non-infectious foot diseases and lesions also contributed. Among infectious foot diseases, OID was the most prevalent (21.3%) followed by IFR (8.0%); WLD and hoof wall cracks were the most prevalent non-infectious foot-lesions (37.7% and 15.3%, respectively). IFR and OID prevalence increased with age (*p* < 0.05) and BCS (*p* < 0.01), respectively, suggesting that host-related factors and husbandry practices are important determinants of its occurrence.

## 1. Introduction

In recent years, the growing demand for sheep milk and products thereof has resulted in a remarkable increase of the global dairy sheep population (ca. 20.0%), and total sheep milk production (ca. 28.0%) [1]. To address this trend, farming systems have been evolving and adapting to more intensive management schemes, exploiting improved genotypes and modern husbandry practices and technologies. This is mainly evident in developed Mediterranean countries with a long-lasting tradition on dairy sheep farming, and a well-organized sheep milk processing industry (e.g., Greece, Spain, France, Italy, etc.).

Intensification of production has reshaped the labor conditions and the socioeconomic status of farmers, converting sheep farming into an attractive career opportunity for young people in rural and peri-urban areas. It has also led to significant benefits for the dairy sheep sector including: (i) increased productivity; (ii) efficient utilization of available resources (such as high-yielding breeding stocks, modernized infrastructures, specialized labor, alternative feedstuff, and optimized land use); (iii) precision farming through adoption of advanced monitoring systems and husbandry practices; and (iv) the establishment of evidence-based biosecurity and hygiene measures for the control of infectious and parasitic diseases [2]. Nevertheless, a growing public skepticism occurs regarding the ethical treatment of animals and their well-being in intensive farming systems [3]. Indeed, several studies have stressed the emergence of health and welfare issues that further influence public perception challenging the sustainability of farms [4,5,6,7]. In addition, heterogeneity of intensive farms in terms of their structure, management, and production methods, minimizes the possibility of universally applicable herd health and disease control protocols [8]. Therefore, an updated, objective, and representative typology of these farms based on their characteristics and animals’ health and welfare status is useful for the assessment of flock health management status and the proposal of targeted modifications to address critical challenges.

Lameness is a condition with considerable impact on dairy sheep productivity, health, and welfare. It is defined as the deviation from normal gait caused by a wide range of factors, usually followed by signs of pain or discomfort and described as a clinical sign rather than a disease itself. The most common causes of lameness include various foot and hoof infections and lesions affecting foot tissues (including joints and bones) that lead to a condition collectively described as foot-related lameness [9]. The major infectious causes of foot-related lameness are either bacterial, namely, infectious footrot (IFR) (*Dichelobacter nodosus* and *Fusobacterium necrophorum*), contagious ovine digital dermatitis (CODD) (*Treponema* spp.), ovine interdigital dermatitis (OID) (*F. necrophorum*), and pedal joint abscess (PJA) (*F. necrophorum* and *Actinomyces pyogenes*), or viral, namely, orf (*Parapoxvirus*), foot and mouth disease (*Apthovirus*), and bluetongue (*Orbivirus*). Non-infectious causes of lameness include white line disease (WLD), laminitis, and other foot lesions and injuries (e.g., toe granulomas, hoof wall cracks, overgrown hooves, foreign bodies, etc.) [10].

Foot-related lameness ranks highly on the list of the most important health issues with the potential to significantly undermine animals’ performance and farms’ sustainability. The reduction on milk yield (ca. 20.0%, ref. [11]), body weight (11.6%, ref. [12]), and wool production (8.0%, ref. [12]) has been documented in lame sheep, explaining the considerable monetary losses derived from foot-related lameness. For example, the annual economic losses in the UK and New Zealand due to lameness have been estimated to reach GBP 24–80 M and NZD 11 M, respectively [13,14].

It is known that both genetic and environmental factors predispose to foot diseases, lesions, and lameness thereof in sheep [9,14,15,16]. For example, polymorphisms of the DQA-2 loci in the ovine Major Histocompatibility Complex (MHC-*ovar*) genes have been associated with the immune response of sheep to IFR, demonstrating a pivotal role in the susceptibility or resistance against the disease [17]. Additionally, hoof conformation and hoof keratin quality are fundamental to maintain the foot health status [16], however, selective breeding for these traits has not been extensively applied. Environmental factors predisposing to foot-related lameness include animal management, nutritional status, housing conditions (bedding moisture, ventilation, temperature, etc.), season and climate, farmers’ knowledge/skills on foot care, and overall hygiene status [18,19]. Other animal factors potentially affecting foot health in sheep are age, productive stage, and milk yield [9]. Nevertheless, little attention has been paid to foot-related lameness in dairy sheep and the available literature is scarce; thereby, much uncertainty still exists regarding the epizootiology and risk factors of lameness-related foot diseases and lesions [9].

A decade ago, in 40% of the studied intensive and semi-intensive Chios dairy sheep farms in Greece, prevalence of foot-related lameness was greater than 5.0% [18], with the prevalent diseases being IFR, OID, PJA, and WLD; since then, farming systems have evolved and foot-related lameness epizootiology is likely to have changed. In meat and wool sheep farms, updated information on foot-related lameness etiology and epizootiology is currently available, underpinning its significance. For example, in the UK, prevalence of specific diseases ranged from <1.0% to >25.0% [20,21], with more than 80.0% of meat sheep flocks, reporting an increase on the occurrence of foot-related lameness [21]. Similarly, in dairy cows, various studies in the UK and the USA place foot-related lameness among the most significant health and welfare issues, with the prevalence ranging from 21.0 to 35.0% [15,22,23,24]. However, extrapolation of epizootiological data from dairy cows or/and meat and wool sheep and applicability of published research in dairy sheep are problematic (for example, in dairy cattle, foot-related lameness is equally caused by infectious and non-infectious lesions unlike dairy sheep where infectious lesions are more frequently observed in lameness cases). This is due to (i) different species and dissimilar productive orientation, husbandry, health management, and breeding practices at the farm level, and (ii) the regional variety of soil-climatic conditions associated with different production methods. Hence, foot-related lameness cannot be evaluated and prioritized on ad hoc basis and subsequently, the suggestion of lameness mitigation strategies is not possible in intensive dairy sheep farms, although an urgent need to cope with the problem is evident.

To address the aforementioned research gap, the objectives of this cross-sectional study were (i) to investigate and describe the epizootiology of lameness-related foot diseases and lesions, and (ii) to assess the impact of potential risk factors on them, in intensive dairy sheep farms in Greece.

## 2. Materials and Methods

### 2.1. Area of the Study

**Figure 1:**
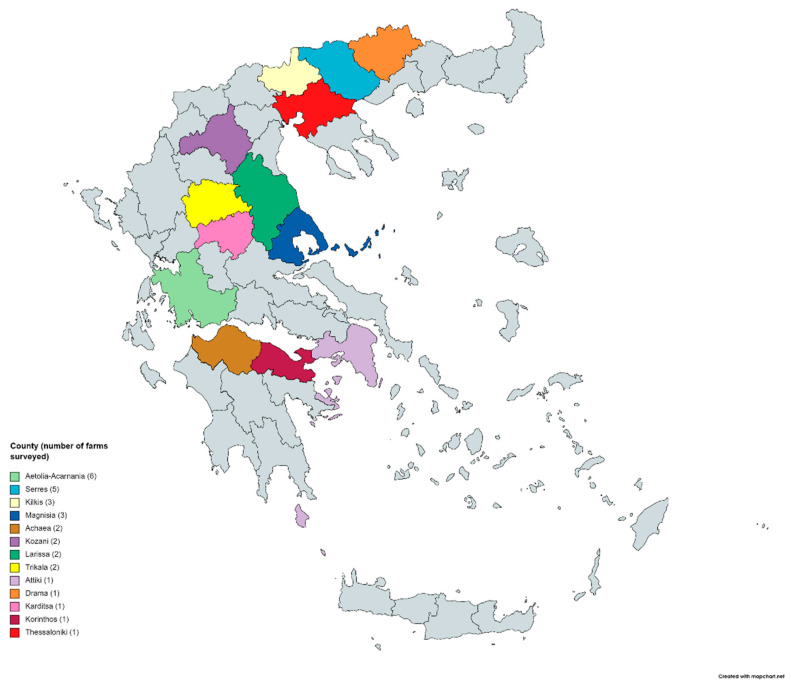
Geographical distribution of the 30 surveyed intensive dairy sheep farms.

Thirty intensive dairy sheep farms (high-input farms with considerable capital investment on breeding stocks, labor and infrastructures) were initially included in the study (n = 10,630 ewes). The farms were distributed in thirteen counties across Greece (Achaea, Aetolia-Acarnania, Attiki, Drama, Karditsa, Kilkis, Korinthos, Kozani, Larissa, Magnisia, Serres, Thessaloniki, Trikala) as presented in Figure 1, and located mainly in plain areas with the topography ranging from coastal areas to inland plateaus and the climate from typical Mediterranean to continental.

Farms were surveyed on-site between May and July 2020, using a structured on-purpose built questionnaire. Data regarding farm structure, flock characteristics and management, labor, infrastructures, feeding and nutrition, reproduction, biosecurity and hygiene measures, disease control protocols, as well as animal overall and foot health status were collected (prevalence, severity, and control measures of lameness-related foot diseases and lesions). A score from 0 to 12 for preventive flock management was calculated assigning one degree for each of the following preventive measures implemented on a regular basis: vaccination against (i) clostridial diseases, (ii) contagious agalactia, (iii) enzootic abortion, (iv) pasteurellosis, (v) gangrenous mastitis, (vi) IFR, (vii) dry-off intramammary antibiotic treatment, (viii) anthelmintic treatment of lambs, (ix) anthelmintic treatment of ewes, (x) vitamin and mineral supplementation, (xi) preventive antibiotic treatment in lambs, and (xii) foot-trimming more than once per year. This score was used as a rough indicator of the preventive veterinary and hygiene status at the flock level.

### 2.2. Farm Selection

A multivariate statistical analysis was used to produce a typology of intensive farming systems by grouping the farms into representative clusters as described in the statistics section below. Based on the results, farms were assigned into two clusters with 22 (Cluster 1) and 8 (Cluster 2) farms, respectively. Three farms per cluster were randomly selected and enrolled in the main study.

### 2.3. Animal Selection and Recording

From each farm, 100 multiparous milking ewes, at the beginning of lactation (20–50 days post-lambing), were randomly selected (total n = 600 ewes). The farms were visited during scheduled routine foot-trimming, which was performed by trained personnel under the supervision of two veterinarians. Before and during foot-trimming, one of the veterinarians clinically assessed and recorded the hoof wall overgrowth and cracks, as well as the occurrence, topography, and severity of foot diseases and lesions including OID, IFR, and WLD at the hoof level. The other veterinarian performed physical examination of the animals and recorded clinical findings and welfare indicators at the animal level. The recorded traits included (i) body condition score (BCS, 1–5, 1 = emaciated, 5 = obese with 0.25 increments) [25], (ii) occurrence of foot-related lameness, arthritis, respiratory disease, ocular and nasal discharge, body abscesses, mastitis, udder lesions and deformities (i.e., skin lesion, abscess, and asymmetry) (0 = absence, 1 = presence), and (iii) wool quality (0 = good quality, 1 = poor quality) and fleece cleanliness (0 = clean, 1 = dirty). Ear tag, breed, and age were also recorded.

### 2.4. Statistical Analyses

**Figure 2:**
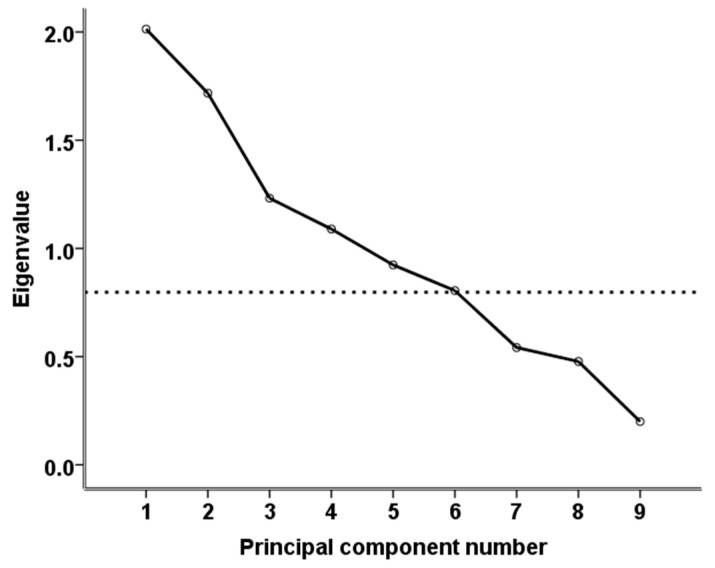
Eigenvalues of the principal components.

SPSS v23 software (IBM Corp., Armonk, NY, USA) was used for the statistical analyses, and statistical significance was set at the 0.05 level. To classify the 30 farms into representative clusters, a two-steps approach was followed. In brief, at the first step, principal component analysis (PCA) was used for data mining purposes, resulting in six principal components (PCs) with eigenvalues greater than 0.8 (Figure 2) to be retained. The six extracted PCs explained about 86.5% of the total variation described by the nine initially considered variables. However, the Kaiser–Meyer–Olkin measure of sampling adequacy was low (0.338) and Bartlett’s test of sphericity was not significant (*p* = 0.186), indicating a poor performance of the PCA; thus, the extracted PCs were not used for the subsequent clustering of the farms. Instead, k-means cluster analysis was used to allocate the farms into two clusters based on factors potentially predisposing to foot-related lameness prevalence (years of farmer’s experience, ewes’ replacement rate, annual milk yield per ewe, prolificacy, stocking density, and annual incidence of foot-related lameness).

Descriptive statistics were estimated (mean ± standard error), whereas comparisons between the two clusters regarding farm characteristics were performed using analysis of variance (ANOVA). Chi-square test (χ^2^ test) was used to compare the prevalence of foot-related lameness and specific non-infectious and infectious foot lesions between (i) the two clusters at the animal-, the limb-, and the hoof level, (ii) front/rear limbs at the limb level, and (iii) front/rear and inner/outer hooves at the hoof level. χ^2^ test was also used for comparisons between the two clusters considering other recorded health and welfare traits.

For the cross-sectional study, all data were integrated into three databases, corresponding to records at the animal-, the limb-, and the hoof- level (600, 2400, and 4800 records, respectively). Prevalence of (i) lameness, and (ii) lameness-related foot diseases and lesions were estimated. To test the contribution of potential risk factors in predicting the occurrence of foot-related lameness, foot diseases and lesions at the animal level, a set of multilevel binary logistic regression models was used, where random effects of farm *j* and animal *i* and fixed effects of age, wool quality, and BCS were considered.

Logit [Pr (Y*_ij_* = 1)] = *β*_0*j*_ + *β*_1_*AGE_ij_ + *β*_2_*WOOL*_ij_* + *β*_3_*BCS*_ij_* + ε*_ij_* (1)

where Y*_ij_* = dependent variable (occurrence of foot-related lameness, OID, IFR, WLD, and hoof wall cracks, on at least one limb), *β*_0*j*_ = intercept, *β*_1_ = coefficient of age (AGE) (4 levels), *β*_2_ = coefficient of wool quality (WOOL) (2 levels), *β*_3_ = coefficient of body condition score (BCS), ε*_ij_* = random residual error.

## 3. Results

### 3.1. Descriptives and Comparisons between the Clusters

Descriptive statistics and comparisons between the two clusters regarding farm and animal characteristics are presented in Table 1. In general, farms in the two clusters had similar structure and characteristics except for (i) milk yield per ewe per lactation which was significantly higher in Cluster 1 (ca. 383 kg) compared to Cluster 2 (ca. 257 kg) (*p* < 0.001), and (ii) number of empty ewes and abortion rate which were significantly higher (*p* < 0.01 and *p* < 0.05, respectively) in Cluster 2 (ca. 10.0% and 4.0%, respectively) compared to Cluster 1 (ca. 4.0% and 1.0%, respectively) (Table 1).

### 3.2. Prevalence of Foot-Related Lameness, Foot Diseases and Lesions at the Animal Level

A total of 210, 98, 230, and 62 ewes of two, three, four, and >four years old, respectively were used for the study. Average BCS of the studied ewes was 2.8 ± 0.01. The overall prevalence of foot-related lameness was 9.0% (54/600), and was significantly higher in Cluster 1 (11.3%, 34/300) compared to Cluster 2 (6.7%, 20/300) [χ^2^ (1, n = 600) = 3.99, *p* < 0.05, Table 2]. The major cause of lameness was IFR (5.5%, 33/600) and its combination with (i) OID (1.2%, 7/600), (ii) WLD (1.0%, 6/600), and (iii) OID and WLD (0.2%, 1/600); other causes of lameness were WLD (0.8%, 5/600) and excessive hoof wall overgrowth (0.3%, 2/600).

**Figure 3:**
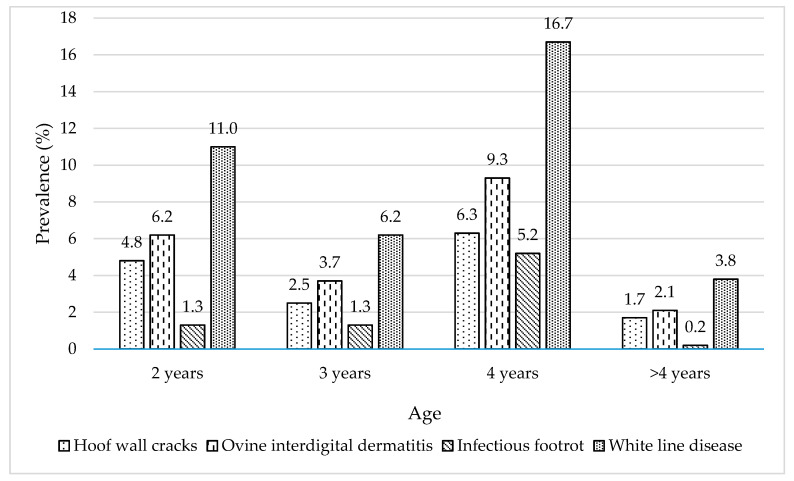
Prevalence of foot diseases and lesions per age group.

In the case of infectious foot diseases, OID was the most prevalent (21.3%, 128/600) followed by IFR (8.0%, 48/600). Regarding non-infectious foot lesions, almost all the studied animals had at least one overgrown hoof (99.3%, 596/600), whereas the prevalence of WLD and hoof wall cracks were 37.7% (226/600) and 15.3% (92/600), respectively. Prevalence of foot diseases and lesions per age group are presented in Figure 3. All foot lesions were observed in each of the studied farms, with OID, IFR, WLD, and hoof wall cracks prevalence ranging from 7.0 to 48.0%, 2.0 to 14.0%, 30.0 to 51.0%, and 13.0% to 18.0%, respectively.

Prevalence of foot lesions, and other health and welfare traits recorded at the animal level, in the two clusters, and comparisons between them are summarized in Table 2; OID prevalence was significantly higher in Cluster 2 (*p* < 0.01); on the contrary, IFR prevalence tended to be higher in Cluster 1 (*p* = 0.071).

### 3.3. Prevalence of Foot Diseases and Lesions at the Limb Level

Prevalence of foot diseases and lesions at the limb level and comparisons between (i) the two clusters, and (ii) front/rear limbs in the studied sheep population, are presented in Table 3. IFR prevalence was significantly higher in Cluster 1 compared to Cluster 2 (*p* = 0.05). Additionally, both OID and IFR prevalence were significantly higher in rear compared to front limbs (*p* < 0.001 and *p* < 0.01, respectively).

**Figure 4:**
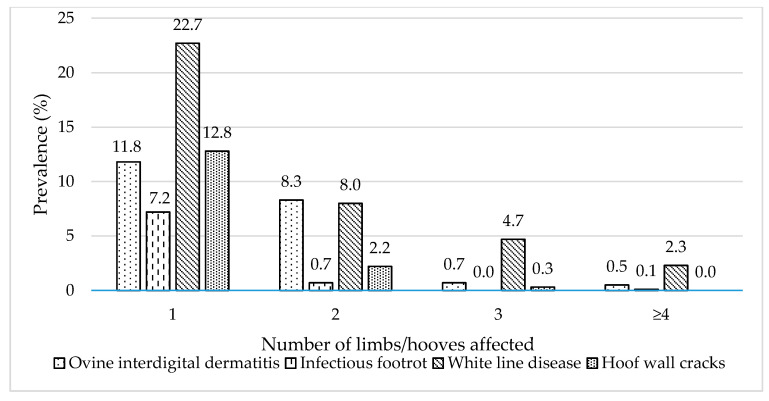
Prevalence of foot diseases and hoof lesions according to the number of the affected (i) limbs (1 to 4) (ovine interdigital dermatitis), and (ii) hooves 1 to ≥4 (infectious footrot, white line disease, hoof wall cracks).

Prevalence of foot diseases and hoof lesions according to the number of the affected (i) limbs (OID), and (ii) hooves (IFR, WLD, and hoof wall cracks) are presented in Figure 4.

### 3.4. Prevalence of Foot Diseases and Lesions at the Hoof Level

Prevalence of IFR, WLD, hoof wall overgrowth, and hoof wall cracks at the hoof level and comparisons between (i) the two clusters, (ii) front/rear hooves, and (iii) inner/outer hooves are presented in Table 4. IFR prevalence at the hoof level was significantly higher in Cluster 1 compared to Cluster 2 farms (*p* < 0.05), and in rear compared to front hooves (*p* < 0.01).

### 3.5. Risk Factors for the Occurrence of Lameness and Lameness-Related Foot Lesions

The effects of potential risk factors on the occurrence of lameness and lameness-related foot diseases and lesions are presented in Table 5. A one-degree increase on BCS (i) tended to be associated with ca. 2.9 times higher likelihood of foot-related lameness (*p* = 0.064), and (ii) was associated with ca. 3.7 times higher likelihood to develop OID lesions (*p* < 0.01). Additionally, ewes with good wool quality were ca. 2.3 times more likely to develop OID lesions (*p* < 0.05) compared to ewes with poor wool quality. Greater than four-year-old ewes were ca. 7.7 and 12.5 times more likely to develop IFR lesions compared to three (*p* = 0.064) and four-year-old (*p* < 0.05) ewes, respectively.

## 4. Discussion

To the best of our knowledge, this is the first study assessing the epizootiology and distribution of foot diseases and lesions in intensively reared dairy sheep in Greece, addressing a significant dearth of knowledge and facilitating better understanding of foot-related lameness; OID and IFR were the most observed infectious foot diseases, whereas WLD and hoof wall cracks were the most common non-infectious hoof lesions. In addition, IFR was the major cause of lameness in the studied flocks.

In our study, a multivariate approach was utilized, integrating potential lameness risk factors as classifying variables to achieve an *a posteriori* typology of intensive farming systems. Similar approaches have been exploited in the past, to classify dairy small ruminant farms in Greece, based on their structure and characteristics, aiming to describe and understand the changes, challenges, and future perspectives of the sector [26,27]. Apart from milk yield and specific reproduction efficiency traits (empty ewes and abortion rate), no other remarkable differences were observed between the two clusters. Therefore, it can be assumed that a common evolutionary pattern exists in intensive dairy sheep farms in Greece; nevertheless, peculiarities of management and husbandry practices are likely to modify productivity and foot-health status. Cluster 1 farms had significantly increased milk yield per ewe per lactation and a higher prevalence of foot-related lameness and IFR compared to Cluster 2; hence, it could be hypothesized that sheep with higher milk yield are more susceptible to foot-related lameness. This is a hypothesis supported by studies in dairy cows showing that high yielding animals are more likely to develop foot lesions and lameness [23,28,29]; however, this is not sufficiently evidenced in dairy sheep [11], and under the current study-design, it is not possible neither to conclude the pathophysiological mechanisms nor estimate the effect of milk yield on foot-related lameness. Cohort studies comparing groups of high- and low-yielding ewes with regard the occurrence of foot-related lameness across lactation are necessary to address this hypothesis. On the contrary, the effect of foot-related lameness on milk yield has been documented in dairy sheep [11,30]. Similarly, in dairy cattle, foot-related lameness was associated with remarkable reduction on daily milk yield (from 1.6 to 2.7 kg/day; [31,32]) and milk yield per lactation period (from 270 to 574 kg [33]).

Overall prevalence of foot-related lameness in the studied ewes population (9.0%) was increased compared to the mean prevalence reported by the farmers during the survey (2.4 and 5.3%, for Cluster 1 and Cluster 2, respectively), and the estimated prevalence in intensive and semi-intensive Chios sheep farms in Greece about a decade ago (6.8%) [30]. In the UK, results regarding prevalence of foot-related lameness are controversial, demonstrating either a reduction [13,34,35] or an increase [21] compared to the average prevalence (8.0–10.0%) reported 15 to 20 years ago [36,37]. The results of our study and the data from the UK indicate that lameness mitigation strategies are not universally efficient; also, we need to be cautious when interpreting the progression of foot-related lameness prevalence, as it is frequently underestimated by the farmers, due to different attitudes and beliefs and the underdiagnosis and underreporting of the problem [38,39]. Interestingly, in our study, foot-related lameness was considered by all farmers as a significant challenge for the health and welfare status of their flock. Nevertheless, most of them could recognize only severely lame animals and major foot diseases and lesions thereof (i.e., IFR, OID but not WLD); this is not consistent with sheep farmers in the UK, who could recognize even mildly lame sheep, although their attribute towards lameness varied [37].

OID was the most frequently observed infectious foot disease with almost one out of five animals in our study being diagnosed with the disease and the prevalence among the flocks varying from 7.0 to 48.0%. Mean prevalence of OID in the UK has been estimated at 6.9% [13], however, prevalence up to 45.0% has been reported [40]. Frequently, OID is considered as the early stage of IFR and defined by infection and exudative inflammation signs at the interdigital space, caused by *F. necrophorum* before the invasion of *D. nodosus* and the underrunning of the hoof [41,42,43]. In our study, OID was separately assessed and defined by the occurrence of mild superficial lesions and loss of hair, limited at the interdigital space, without the occurrence of underrunning of the hoof wall. In general, early stages of OID either are not followed by lameness, or result in unnoticed lameness cases [43]; this is consistent with our findings where mild to moderate OID cases were observed and not found to be associated with lameness unless IFR co-existed. Absence of lameness indicates that OID is possible to remain underdiagnosed or undiagnosed in intensive sheep farms until its complication by *D. nodosus* and the development of IFR. Furthermore, OID was more commonly observed at the rear limbs compared to the front limbs. According to the available literature, this is the first time that differences between front and rear limbs regarding OID infections are observed in sheep. However, this is in agreement with the distribution of lesions due to infectious foot diseases in both dairy cows and beef cattle [23,44,45,46]. In the case of dairy cows, it has been suggested that the closer contact of rear limbs with manure (*F. necrophorum* is normally found on the digestive tract), and potentially body weight distribution, may explain the susceptibility of rear limbs to the disease. Nevertheless, in sheep farms, manure and bedding material are drier than in cow farms, whereas research on weight distribution and posture/gait biomechanics is scarce; hence, support or rejection of these hypotheses are not feasible.

IFR is a globally spread and exceptionally contagious and painful disease of the foot. It is considered the most significant lameness-related bacterial foot disease in sheep causing extended foot lesions [47]. These lesions include the underrunning of the hoof, and in advanced cases, the total separation of the horn from the underlying hoof matrix [43,48,49]. Its prevalence in meat sheep varies from negligible (0.4%, [50]) or moderate (3.7% [37]; 8.5% [51]) to high (>40.0% [52]) or extremely high (>95.0% [43]) and its transmission occurs mainly in damp conditions, rather than in dry, hot, and cold conditions [53]. Under the UK temperate climate, 80.0 to 90.0% of sheep flocks are affected by IFR [21,54], with the annual cost of the disease estimated between GBP 20 to 80 million [55,56]. In our study, IFR was observed in all the studied flocks, and 8.0% of the studied ewes were affected by IFR in at least one hoof; this is within the expected prevalence range, considering that recordings took place in autumn, when warm weather and high moisture favor survival, proliferation, and transmission of *F. necrophorum* and *D. nodosus* [9,42,49]. IFR was present in 87.0% (47/54) of the ewes with lameness alone or in combination with other foot lesions. This finding underlines its significance in the epizootiology of foot-related lameness in intensive dairy sheep farms, which is in agreement with Gelasakis et al. [30] and Kaler and Green [37] who documented that more than 60.0 and 90.0% of lameness cases were due to IFR in dairy and meat sheep, respectively. IFR had a significantly higher prevalence in cluster 1 ewes, when examined at the limb and the hoof level, implying an association between high milk yield and susceptibility to IFR, which is consistent with findings in dairy cows [28]. IFR was more commonly observed at rear limbs and hooves compared to front limbs and hooves, possibly explained by the mechanism detailed for OID. As expected, and in compliance with the available literature, no significant differences were found between inner and outer hooves as regards to the occurrence of the disease.

The most prevalent foot lesion in the studied sheep population was WLD (ca. 38.0%). High prevalence of WLD is not unusual. Previous studies have reported prevalence as high as 75.0% in some flocks [10,57]. WLD occurs when the hoof wall is detached from the laminar corium, a condition that is usually underdiagnosed or under-reported by the farmers who are unable to recognize it. This was also the case in the surveyed farms. Thereby, high prevalence of WLD could be associated with misdiagnosis and the absence of efficient treatment. In most cases, WLD is not directly associated to lameness, but predisposes to it due to debris accumulation inside the defect, leading to proliferation of bacteria, foot infection, abscess formation (WLA), pain and lameness. Although, the etiology and risk factors of WLD remain unknown in sheep [10], type of ground [58] and nutritional deficiencies (biotin and sulfur containing amino acids) are considered as causative or predisposing factors in dairy cattle [59]. Moreover, genetic background has recently been evidenced for WLD in meat sheep breeds using data from 9169 sheep and 22 farms with increased WLD prevalence (47.0% and 24.0%, for Scottish Blackface and Texel sheep, respectively) in the UK [57]. In dairy sheep, data regarding the epizootiology of WLD is limited. Recently, Gelasakis et al. [30] reported white line lesions (WLD and WLA) as the second most significant cause of foot-related lameness in intensively reared Chios sheep (with an incidence risk of 1.1% and 0.8%, respectively); however, only lame animals were included in that study. Regarding the distribution of WLD lesions at the limb and the hoof level, no significant differences were found, which agrees with findings from dairy goats in the UK [60]; hence, it could be assumed that WLD is not likely to be associated with (i) body weight distribution and biomechanics during standing and walking, (ii) anatomical peculiarities of hooves, and (iii) different environmental exposures, at the limb or the hoof level (e.g., associated with their placement, front/rear or inner/outer). In the present study, none of the factors assessed was found to affect the prevalence of WLD. Breed has been found to predispose in WLD in both meat sheep and dairy cows, whereas WLD prevalence in cows increased with age and milk yield [58,61]. Although we used only purebred animals of different breeds, the breed effect could not be estimated as it was confounded by the farm effect. When assessed at the hoof level, WLD tended to be most prevalent in Cluster 2 ewes (*p* = 0.06), indicating that differences between intensive farming systems regarding WLD prevalence are likely to occur. In every case, it can be concluded that a different study-design (e.g., cohort study) should be exploited to investigate the etiopathogenesis and epizootiology of WLD.

This is the first time hoof wall cracks in dairy sheep are studied, whereas relevant studies in meat and wool sheep are also not available. Hoof wall cracks were observed in ca. 15.0% of the ewes. Based on horse and cattle studies, inappropriate housing conditions, poor bedding, increased moisture, manure exposition, vitamin and trace mineral deficiencies, poor-quality hoof keratin and hoof conformation, as well as increased age and BCS lead to the disruption of hoof wall integrity and may predispose to hoof wall cracks and injuries [62,63,64,65,66]. Poor wool quality could be linked to a higher prevalence of hoof wall cracks, due to the fact that keratin of both wool and hoof horn have similar low- and high- sulfur protein fractions [67,68,69]; however, this was not confirmed by the model applied in our study. In dairy cows, a much lower prevalence (1.0%) of hoof wall cracks has been found, compared to beef cows (64.0%), mainly in the outer, front hooves associated with the gait pattern and the charging of the front limbs [66,70]. In our study, no significant differences were found between the clusters, front/rear limbs and hooves, and inner/outer hooves, whereas none of the traits assessed as risk factors had a significant effect.

Inappropriate foot care (i.e., foot-trimming and foot-bathing), also predisposes to foot-related lameness. Indeed, severe hoof overgrowth may cause foot deformities and is likely to predispose to infectious foot diseases, foot lesions, and lameness [71]. Τhe majority of farms in the present study exercised routine foot-trimming once per year. Foot baths are rarely used, if at all. As expected, overgrown hooves were observed in almost all the studied animals regardless of cluster or farm. For this reason, the observed differences regarding hoof overgrowth at the limb and the hoof level are biologically meaningless. In dairy cows, differences between front and rear hooves regarding prevalence and severity of hoof overgrowth have been documented, with front hooves being more frequently severely overgrown [72]. In permanently housed dairy sheep, hooves are not worn-down. Animals usually walk on soft straw bedding material which does not favor natural wearing of the hoof. In addition, increased intake of concentrates in high yielding sheep further deteriorates the situation by increasing the growth rate of the hoof horn [73]. In the studied farms, excessive hoof overgrowth implies that routine foot-trimming once per year was not adequate, hence, twice per year may be necessary (i) to maintain normal shape and conformation of the hooves and the uninterrupted movement of the animals, (ii) to avoid injuries and infections, and (iii) for the early diagnosis and allocation of foot-lesions. In every case, it is crucial that foot-trimming is performed by experienced foot-trimmers to avoid excessive trimming leading to injuries and bleeding. According to Winter et al. [13], the remarkable decrease in the prevalence of sheep lameness in England from 2004 to 2013 in meat breeds was linked to (i) the control of excessive foot-trimming, (ii) the prompt treatment of lameness, and (iii) the overall training and knowledge-transfer to farmers for the management of lameness. Based on this observation, they concluded that foot-trimming should be excluded from routine foot care practices, as its benefits were not evidenced. However, extrapolating observations from meat sheep, reared under grazing systems where natural worn-down of the hooves occurs, is not appropriate.

Season is among the environmental factors affecting foot-related lameness in both meat [74] and dairy sheep [9]. Our study was performed between mid to late autumn (from October to December), where increased moisture and relatively warm weather in combination with accumulating manure facilitate the proliferation and transmission of pathogens causing infectious foot diseases, thus justifying the observed high prevalence of OID and IFR [42,74]. Under the current study-design, it was not possible to assess the seasonal variation of infectious foot diseases prevalence, and a longitudinal study is warranted to address this objective. A similar study was recently implemented in meat sheep, demonstrating increased prevalence of IFR during late summer/early autumn and spring [74].

Farming system influences the etiology and epizootiology of foot-related lameness in dairy sheep [9], with intensive farms being more challenged [39]. In intensive farms, increased stocking density [13] and inappropriate hygiene conditions [75] predispose to foot diseases and lameness thereof. Hence, increased stocking density in Cluster 2 could explain the significantly higher prevalence of OID, ocular and/or nasal discharge, mastitis, and udder skin lesions, as well as the dirtier fleece of the ewes. Noteworthy differences regarding basic hygiene and biosecurity protocols were not recorded among the farms; routine cleaning and disinfection of the premises, with commercial disinfectants, at least twice per year, during early autumn and spring were practiced in all cases following standard procedures.

Average BCS of the studied ewes was acceptable (2.8) for the production stage (early lactation). Ideally, BCS during early lactation should be 3.0, however, negative energy balance at this stage in high-yielding dairy ewes is common resulting in a rather expected reduction on BCS during the first weeks of lactation [76]. Ewes with higher BCS were more likely to be diagnosed with OID. It is the first time such a relationship is observed in dairy sheep, although similar observations have been previously reported in dairy cows where heavier animals had increased probability of developing non-infectious hoof disorders [77]. Similarly, ewes with better wool quality in the present study had a higher risk of being infected by OID. Most OID cases were mild and not complicated by IFR; it can be hypothesized that ewes with better wool quality and/or higher BCS had a better quality of hoof horn preventing the transition from OID to IFR and lameness.

In the studied flocks, distribution of animals within the four age groups was not equal, although animals were randomly selected. Nationwide drop of sheep milk price by 20.0 to 25.0% in 2017–2018 resulted in a wide reduction of the replacement rate that year, to decrease replacement cost. This was also the case in the studied flocks, leading to a reduced three-year-old age group. Random selection of animals and analyses performed were adequate to handle and compare data between unequally sized age groups. Greater than four-year-old ewes had significantly increased probabilities of developing IFR compared to three- and four-year-old ewes. This is consistent with several studies in meat sheep [16,43,74], and is probably the result of increased susceptibility to footrot and hoof deterioration with age [16]. This is also supported by several studies in dairy cows [78,79,80,81,82,83], where increased prevalence of infectious foot-diseases with age is attributed to (i) hoof deformities associated with metabolic and other stressors [78,84], (ii) changes in the loading capacity of the sole and other soft tissues of the feet [85], (iii) longer exposure to housing environment and pathogens [78], and (iv) inadequate treatment and relapsing foot lesions [81].

## 5. Conclusions

A remarkable prevalence of foot-related lameness, foot diseases and lesions in intensive dairy sheep farms in Greece has been evidenced, indicating an increasing trend over the last decade. This underpins the urgent demand for targeted mitigation strategies against foot-health challenging conditions and particularly infectious foot diseases (i.e., IFR and OID). These strategies need to adjust on the peculiarities of intensive livestock farming and should be mainly based on (i) hygiene and biosecurity measures, (ii) preventive veterinary measures and control of foot diseases and lesions, and (iii) training of farmers on foot care practices and holistic management of foot health and lameness. Among the studied risk factors, age, and BCS were associated with infectious foot diseases; however, further investigation of potential risk factors in cohort and large-scale studies are crucial to elucidate their effects. Building on current knowledge and covering of the gaps in the epizootiology and potential risk factors of foot-related lameness, diseases, and lesions will further enhance health and welfare of dairy sheep and the sustainability of farms.

## Figures and Tables

**Table 1:** Descriptive statistics (mean ± standard error) and comparisons between the two clusters regarding farm and animal characteristics.

	Cluster 1 (n = 22)	Cluster 2 (n = 8)	*p*-Value
Farmers’ experience (years)	10.0 ± 1.36	8.5 ± 1.98	0.561
Animals per employee (n)	159.2 ± 11.21	128.1 ± 17.00	0.155
Total animal number (n)	518.6 ± 98.00	376.8 ± 84.47	0.415
Early lambing ewes (n)	208.6 ± 42.60	137.4 ± 28.87	0.339
Late lambing ewes (n)	167.5 ± 43.86	104.5 ± 35.89	0.417
Rams (n)	15.9 ± 2.04	13.3 ± 3.37	0.506
Ewes to ram ratio	27.6 ± 3.80	23.4 ± 2.96	0.524
Rams’ replacement (years)	3.0 ± 0.27	3.6 ± 0.46	0.247
Replacement rate (%)	24.6 ± 2.35	23.1 ± 4.11	0.746
Milk yield/ewe/lactation (kg)	383.4 ± 8.76	256.9 ± 16.88	0.000
Prolificacy (lambs/ewe)	1.6 ± 0.10	1.5 ± 0.11	0.360
Weaning age (days)	38.6 ± 1.84	40.0 ± 3.13	0.698
Lamb carcass weight at weaning (kg)	10.1 ± 0.48	10.1 ± 0.55	0.939
Ewes per ram at mating	12.6 ± 2.24	15.6 ± 2.95	0.469
Empty ewes (%)	3.9 ± 0.81	10.4 ± 2.67	0.004
Abortion rate (%)	1.2 ± 0.28	2.9 ± 0.88	0.021
Diarrhea rate in lambs (%)	32.6 ± 8.15	43.3 ± 11.31	0.487
Mastitis rate (%)	4.0 ± 1.15	4.1 ± 1.11	0.951
Foot-related lameness rate (%)	2.4 ± 0.50	5.3 ± 2.31	0.085
Stocking density (m^2^/ewe)	2.1 ± 0.13	1.7 ± 0.11	0.083
Preventive flock management score (0–12)	6.1 ± 1.82	5.6 ± 1.77	0.538

**Table 2:** Prevalence of foot lesions (OID: Ovine interdigital dermatitis, IFR: Infectious footrot, WLD: White line disease) and other health and welfare traits recorded during clinical examination at the animal level in the two clusters, and comparisons between them.

	Prevalence, % (n)	Prevalence, % (n)	*p*-Value
	**Cluster 1 (n = 300 ewes)**	**Cluster 2 (n = 300 ewes)**	
Lameness	11.3% (34)	6.7% (20)	0.046
OID	16.7% (50)	26.0% (78)	0.005
IFR	10.0% (30)	6.0% (18)	0.071
WLD	35.0% (105)	40.3 (121)	0.178
Hoof overgrowth	99.7% (299)	99.0% (297)	0.316
Hoof wall cracks	8.0% (48)	7.3% (44)	0.650
Arthritis	3.3% (10)	5.7% (17)	0.168
Poor wool quality	11.7% (35)	6.0% (18)	0.014
Dirty fleece	58.0% (174)	67.0% (201)	0.023
Respiratory sound	1.3% (4)	2.7% (8)	0.243
Ocular/nasal discharge	0.3% (1)	2.7% (8)	0.019
Clinical mastitis	0.3% (1)	3.0% (9)	0.011
Udder abscess	13.7% (41)	17.3% (52)	0.215
Udder skin lesions	1.0% (3)	14.7% (44)	0.000
Udder asymmetry	31.7% (95)	25.0% (75)	0.070
Body abscess	15.3% (46)	19.0% (57)	0.234

**Table 3:** Prevalence of foot lesions (OID: Ovine interdigital dermatitis IFR: Infectious footrot WLD: White line disease) at the limb level (n = 2400 limbs) and comparisons between (i) the two clusters, and (ii) front/rear limbs in the studied sheep population.

		Prevalence, % (n)	Prevalence, % (n)	*p*-Value
Clusters		**Cluster 1 (n = 1200)**	**Cluster 2 (n = 1200)**	
	OID	8.6% (103)	7.7% (92)	0.411
	IFR	2.8% (33)	1.6% (19)	0.050
	WLD	12.5% (150)	14.7% (176)	0.121
	Hoof overgrowth	98.8% (1185)	96.3% (1155)	0.000
	Hoof wall cracks	4.4% (53)	4.3% (51)	0.841
Front/Rear limbs		**Front limbs (n = 1200)**	**Rear limbs (n = 1200)**	
	OID	4.2% (50)	12.1% (145)	0.000
	IFR	1.3% (16)	3.0% (36)	0.005
	WLD	13.4% (161)	13.8% (165)	0.812
	Hoof overgrowth	96.9% (1163)	98.1% (1177)	0.067
	Hoof wall cracks	4.3% (52)	4.3% (52)	1.000

**Table 4:** Prevalence of foot lesions (IFR: Infectious footrot, WLD: White line disease) at the hoof level (n = 4800 hooves) and comparisons between (i) the two clusters, (ii) front/rear hooves, and (iii) inner/outer hooves in the studied sheep population.

		Prevalence, % (n)	Prevalence, % (n)	*p*-Value
Clusters		**Cluster 1 (n = 2400)**	**Cluster 2 (n = 2400)**	
	IFR	1.5% (36)	0.8% (20)	0.032
	WLD	7.1% (171)	8.6% (206)	0.060
	Hoof overgrowth	96.2% (2308)	93.6% (2247)	0.000
	Hoof wall cracks	2.3% (55)	2.3% (54)	0.923
Front/Rear hooves		**Front hooves (n = 2400)**	**Rear hooves (n = 2400)**	
	IFR	0.7% (17)	1.6% (39)	0.003
	WLD	7.7% (185)	8.0% (192)	0.707
	Hoof overgrowth	93.4% (2242)	96.4% (2313)	0.000
	Hoof wall cracks	2.3% (54)	2.3% (55)	0.923
Inner/Outer hooves		**Inner hooves (n = 2400)**	**Outer hooves (n = 2400)**	
	IFR	1.3% (32)	1.0% (24)	0.282
	WLD	7.7% (184)	8.0% (193)	0.629
	Hoof overgrowth	95.8% (2298)	94.0% (2257)	0.007
	Hoof wall cracks	2.2% (52)	2.4% (57)	0.628

**Table 5:** Associations between (i) age, (ii) wool quality, (iii) body condition score and foot-related lameness and foot lesions at the animal level in the 600 studied ewes.

						95 % CI of OR	
Parameter	Category Level	*B*	SE	*p*-Value	OR	Lower	Upper
**Foot-related lameness**							
Age (years)	2	0.39	0.569	0.492	1.48	0.48	4.52
	3	0.23	0.609	0.708	1.26	0.38	4.16
	4	−0.16	0.538	0.767	0.85	0.30	2.45
	>4	*“Ref”*					
Wool quality	Good	−1.68	1.042	0.109	0.19	0.02	1.45
	Poor	*“Ref”*					
Body condition score	Continuous	1.07	0.578	0.064	2.93	0.94	9.10
Intercept	Continuous	0.94	1.909	0.624	2.55	0.06	108.44
**Ovine interdigital dermatitis**							
Age (years)	2	−0.27	0.411	0.520	0.77	0.34	1.72
	3	−0.64	0.448	0.154	0.53	0.22	1.27
	4	−0.44	0.381	0.248	0.64	0.31	1.36
	>4	*“Ref”*					
Wool quality	Good	0.81	0.342	0.018	2.25	1.15	4.40
	Poor	*“Ref”*					
Body condition score	Continuous	1.31	0.431	0.002	3.72	1.60	8.67
Intercept	Continuous	−2.57	1.272	0.044	0.08	0.01	0.93
**Infectious footrot**							
Age (years)	2	−1.27	1.096	0.248	0.28	0.03	2.43
	3	−2.04	1.101	0.064	0.13	0.02	1.13
	4	−2.49	1.039	0.017	0.08	0.01	0.64
	>4	*“Ref”*					
Wool quality	Good	0.37	0.512	0.476	1.44	0.53	3.93
	Poor	*“Ref”*					
Body condition score	Continuous	−0.10	0.606	0.869	0.91	0.28	2.98
Intercept	Continuous	4.48	2.027	0.028	88.11	1.64	4721.07
**White line disease**							
Age (years)	2	0.34	0.323	0.295	1.40	0.74	2.64
	3	0.03	0.352	0.943	1.03	0.51	2.05
	4	−0.19	0.305	0.540	0.83	0.46	1.51
	>4	*“Ref”*					
Wool quality	Good	0.16	0.311	0.617	1.17	0.63	2.15
	Poor	*“Ref”*					
Body condition score	Continuous	0.04	0.308	0.893	1.04	0.57	1.91
Intercept	Continuous	0.20	0.922	0.825	1.23	0.20	7.49
**Hoof wall cracks**							
Age (years)	2	0.09	0.412	0.820	1.10	0.49	2.47
	3	−0.03	0.456	0.956	0.98	0.40	2.39
	4	−0.06	0.394	0.880	0.94	0.44	2.04
	>4	*“Ref”*					
Wool quality	Good	0.19	0.386	0.623	1.21	0.57	2.58
	Poor	*“Ref”*					
Body condition score	Continuous	0.52	0.404	0.203	1.67	0.76	3.70
Intercept	Continuous	0.09	1.171	0.941	1.09	0.11	10.89

OR: Odds Ratio; CI: Confidence interval; *B*: Coefficient; SE: Standard error; *“Ref”*: Reference category.
